# Editorial: Genetics of reproduction for livestock species

**DOI:** 10.3389/fgene.2023.1210904

**Published:** 2023-05-22

**Authors:** Pouya Zamani, Ramin Abdoli, Mohammad Hossein Ferdosi, Shahin Eghbalsaied

**Affiliations:** ^1^ Department of Animal Science, Faculty of Agriculture, Bu-Ali Sina University, Hamedan, Iran; ^2^ Iran Silk Research Center, Agricultural Research, Education and Extension Organization (AREEO), Gilan, Iran; ^3^ Animal Genetics and Breeding Unit, A Joint Venture of NEW Department of Primary Industries and University of New England, Armidale, NSW, Australia; ^4^ School of BioSciences, Faculty of Science, University of Melbourne, Parkville, VIC, Australia; ^5^ Department of Animal Science, Isfahan branch, Islamic Azad University, Tehran, Iran

**Keywords:** farm animals, SNP, litter size, fecundity, CRISPR, GWAS

## Introduction

Livestock farming provides a major source of animal protein and occupation opportunities for a large proportion of the world’s population, and its profitability could be effectively increased by improvement of either feed efficiency (Zamani, 2017) or reproductive performance (Abdoli et al., 2016). Therefore, genetic improvement of reproductive efficiency is an important objective for animal production industries. Reproduction is a complex biological process with low to medium heritability, which indicates significant influences of environmental and non-additive genetic effects on reproductive performance (Zamani and Abdoli, 2019). Because of the low heritability of reproduction traits, classic selection methods are generally inefficient to achieve rapid genetic progression of reproduction performance in livestock species (Abdoli et al., 2019). However, the use of genetic markers may efficiently enhance the selection response of reproduction traits (Abdoli et al., 2016). Regarding the polygenic nature of reproduction traits, the determination of genetic markers and genetic pathways involved in reproduction efficiency needs intensive molecular genetic studies and use of high-throughput technologies, including genome-wide association studies, whole-genome sequencing, and whole transcriptome analysis.

This Research Topic aimed to collect new research in the field of “Genetics of reproduction in livestock species”. After announcing the Research Topic, a total of 20 original articles were received. However, after intensive review processes, only five articles were accepted and published in this Research Topic. A summary of the results of the articles in this Research Topic is as follows.

## The articles

1. Identification of genes related to sexual differentiation and sterility in embryonic gonads of mule ducks by transcriptome analysis (Yang et al.).

Gonad development is a factor involved in the reproduction process in different livestock species. This study was conducted to investigate the genes associated with early-stage gonad development in ducks. Embryonic gonadal tissues of three duck types, comprising mule, Jinding, and Muscovy, were collected and subjected to RNA sequencing analysis. In this study, among a total of 9,471 detected genes, 12 genes related to testicular development, 11 genes highly expressed in female gonads, seven genes responsible for female sterility and eight genes for male sterility, and five genes for both female and male sterility were identified. This study provides new information about the differential development of male and female gonads and sterility mechanisms in mule ducks and a theoretical basis for future research on sex determination and differentiation in ducks, and possibly sterility in birds.

2. Genetic parameters estimation and genome molecular marker identification for gestation length in pigs (Shi et al.)

Pig is a major component of meat production in many countries. Regarding the important role of gestation length in piglet maturation and body development, the aim of this study was the estimation of genetic parameters and identification of genomic molecular markers for gestation length in pigs. In this study estimates of heritability and repeatability for gestation length were 0.16 and 0.24, respectively. Genotyping of animals using the Porcine 50 K Chip and genome-wide association analysis resulted in identification of 1,002 single nucleotide polymorphisms, associated with gestation length. This study proposed 4,588 candidate genes; however, a total of 20 genes were most suggestive of candidate genes. This article provides new genetic parameters and molecular information for gestation length in pigs.

3. Genome-wide analysis for the melatonin trait associated genes and SNPs in dairy goat (*Capra hircus*) as the molecular breeding markers (Wu et al.)

Melatonin is one of the factors regulating reproduction in vertebrates with a strong influence on biological rhythms in many organisms (Talpur et al., 2018). In this study, whole genome resequencing bulked segregant analysis on dairy goats identified a total of 34,921 SNPs relating to 1,177 genes. However, three SNPs, located within exon regions of *ASMT* and *MT2* genes, were significantly correlated with melatonin levels, whereby these SNPs resulted in approximately five-fold higher melatonin levels in blood serum and milk. The results of this study suggest that the three detected SNPs could be used as molecular markers in breeding programs for dairy goats.

4. Comparative gonad transcriptome analysis in cobia (*Rachycentron canadum*) (Shen et al.)

Cobia is known as a fast-growing fish with a high commercial value, and many fish producers favor a monosex culture because of the higher body size of female fish. However, the effects of genetic factors associated with the sexual development and sexual phenotype in cobia are largely unknown. This study is possibly the first attempt to detect sex-biased differentially expressed genes (DEGs) in cobia fish gonads, using comparative gonad transcriptome sequencing analysis. A total of 76 candidate genes, including 42 known testis-biased and 34 known ovary-biased DEGs were identified as the genes involved in sex determination cascades or sex differentiation pathways. A total of 11 pathways, including Wnt signaling, oocyte meiosis, TGF-β signaling, and MAPK signaling pathways, were identified as functionally related to sex determination and differentiation. The results of this study provide a molecular basis for further studies on cobia’s sex determination mechanism and facilitation of monosex culturing.

5. The alternative transcription and expression characterization of *Dmc1* in autotetraploid *Carassius auratus* (Xu et al.)

The different fertilities and meiosis stabilities of established and neo-autotetraploids are known (Yant et al., 2013). This study was conducted to investigate the transcription and expression characterization of *Dmc1* gene (DNA meiotic recombinase 1) in autotetraploid goldfish. This study showed different expressions of *Dmc1* in diploid and tetraploid goldfish, and specific expression of the duplicated *Dmc1* in the autotetraploid individuals is probably involved in meiosis progression of diploid-like chromosome pairing.

## Other interesting areas for study

In addition to the subjects published in this Research Topic, there are several other interesting areas, including high-throughput methods, that should be subjected to future studies on the genetics of reproduction.

### Genome-wide association studies

Genome-wide association studies (GWAS) are widely used for detection of genomic regions associated with productive and reproductive traits. Previous GWASs resulted in detection of many quantitative trait loci and candidate genes associated with reproductive traits in different livestock species, such as cattle (Mohammadi et al., 2022), sheep (Abdoli et al., 2018), goats (Mahmoudi et al., 2022), Poultry (Wolc et al., 2014), and fish (Ambrosio et al., 2020). However, the GWAS results are largely dependent on different factors such as sampling method, linkage disequilibrium, and genetic structure of the studied population. Therefore, more studies are needed to discover other genetic variations of reproduction efficiency in different breeds of livestock species.

### Gene editing

The recent discovery of the CRISPR/Cas9 system has made on-target genetic engineering achievable in any organism. Specific mutations in major genes associated with the performance of farm animals have been a hot Research Topic for more than two decades. This has provided an important phenotypic and genotypic database to search for the most important major genes and mutations. However, instead of successive generations of selection and planned mating, use of a reverse engineering approach can create desired mutation(s) in a single generation by using the CRISPR/Cas9 approach. This technology has been used for the identification of three well-known major fecundity genes in farm animals, including the Booroola mutation in ovine *BMPR1B* (Zhou et al., 2018), the *BMP15* knockout in goats (Tripathi et al., 2021), and the FecGF mutation in ovine *GDF9* (Xu et al., 2023), as well as the targeted deletion of octamer-binding transcription factor 4 (*OCT4*) gene in cattle (Kasimanickam, 2021). However, there are limited numbers of laboratories working on genetic engineering of farm animals compared to those on mice and rats.

### Gene drive

Gene drive comprises the introduction of synthetic fragments involving a driving element, the CRISPR/Cas9 system, into the target genome, which can increase the spreading chance of specific alleles through the population compared to the standard Mendelian inheritance (Friedman et al., 2020). A main application of the genes was eradication of mosquitos to control the spread of malaria or other invasive species on isolated land. Most of the gene drive strategies are based on weakening the fitness of a sex and making a unisex species, all-male for example. Therefore, key elements for sex differentiation are targeted, such as the aromatase gene, in which the knockout females are infertile while the knockout males can still remain fertile and spread the gene drive allele. Another strategy is known as the X-shredder approach, by which the gRNAs target only the X chromosome. Expression of the gene drive system during the gametogenesis phase can degrade all sperm cells carrying the X chromosome and allow only the Y chromosome-bearing sperm cells to function (Galizi et al., 2016). However, the low rate of homology-directed repair (HDR) in mammalian species has been the main challenge that has yet to be tackled efficiently (Eghbalsaied and Kues, 2023). Moreover, because the gene drive element can remain in the genome and spread through the population, there is a high risk for halting or reversing the gene-drive animals and also controlling the genetically modified animals to avoid crossing with other populations that are not considered invasive in other countries and continents (Bier, 2022). This controllability issue is more challenging in low-threshold gene drive systems. Although the modified gene spreads quickly through a population, combining the gene drive system with drive-neutralization elements, such as anti-Cas9 gRNA that imperfectly inactivate the Cas9 protein is suggested to improve the gene drive controllability (Backus and Delbourne, 2019). However, the functionality and efficiency of these systems should be tested in different animals and ecological niches as well.

## Conclusion

Understanding major genes/mutations associated with the reproductive performance of farm animals could have a great impact on the economic efficiency of animal production enterprises. The articles published in this Research Topic improved our understanding of the genetics of reproduction in livestock species. However, there are huge numbers of questions about genetic factors associated with reproduction performance in farm animals, which should be considered in future studies.
